# Supplementary material for: Seminoma and Embryonal Carcinoma Footprints Identified by Analysis of Integrated Genome-Wide Epigenetic and Expression Profiles of Germ Cell Cancer Cell Lines
Source: PLoS One. 2014 Jun 2;9(6):e98330. doi: 10.1371/journal.pone.0098330 (PMC4041891; doi:10.1371/journal.pone.0098330)
Supplement: File S1 — ZIP file containing DMRforPairs output for significant regions. Please start from the html files. (ZIP) [file pone.0098330.s008.zip › figures/27766.pdf]

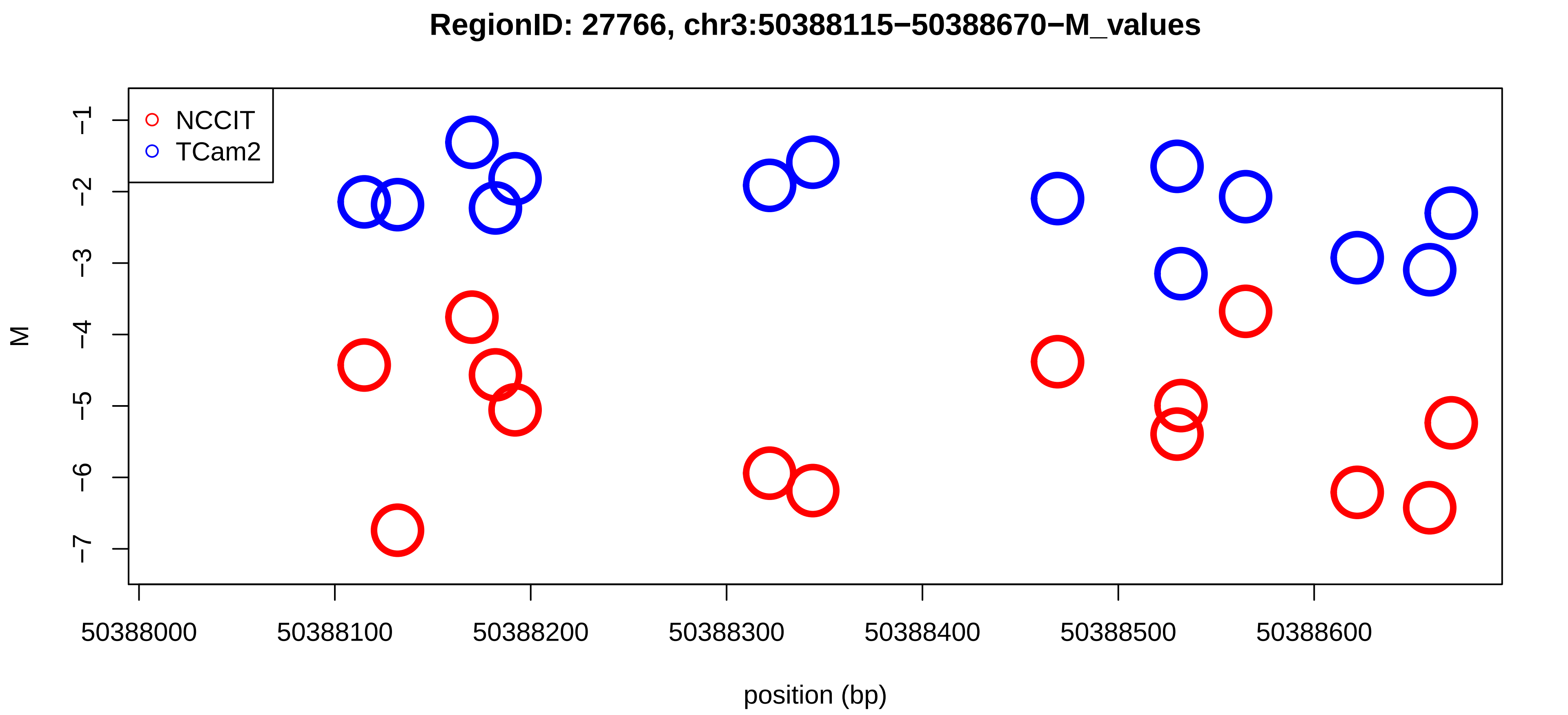
RegionID: 27766, chr3:50388115−50388670−M_values
NCCIT
TCam2
M
−1
−2
−3
−4
−5
−6
−7
50388000
50388100
50388200
50388300
50388400
50388500
50388600
position (bp)

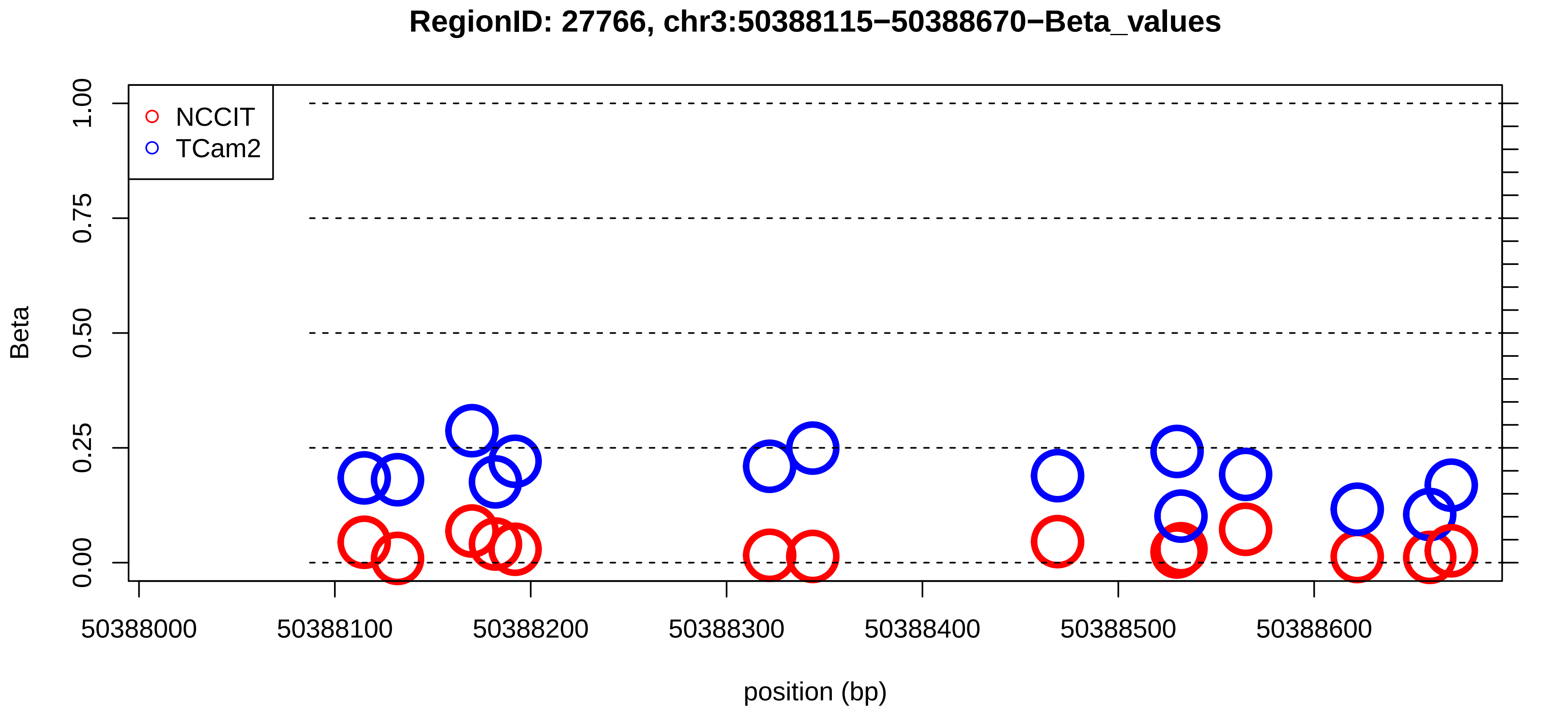
RegionID: 27766, chr3:50388115−50388670−Beta_values
NCCIT
TCam2
Beta
0.00
0.25
0.50
0.75
1.00
50388000
50388100
50388200
50388300
50388400
50388500
50388600
position (bp)

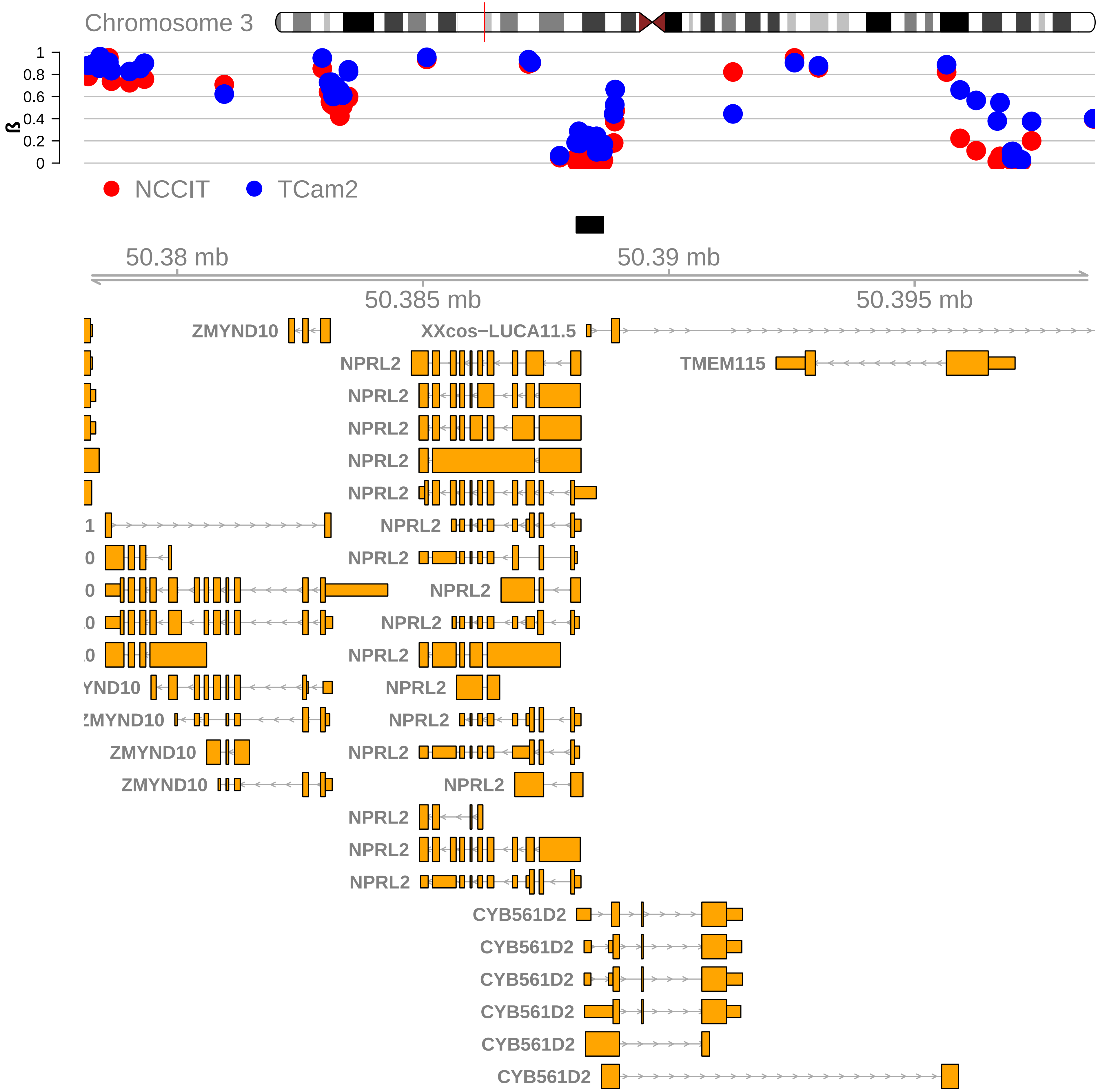
Chromosome 3
ß
1
0.8
0.6
0.4
0.2
0
NCCIT
TCam2
50.38 mb
50.385 mb
50.39 mb
50.395 mb
ZMYND10
XXcos−LUCA11.5
NPRL2
TMEM115
CYB561D2
